# Management of spastic diplegic cerebral palsy using ayurveda

**DOI:** 10.6026/973206300221760

**Published:** 2026-03-31

**Authors:** Ranjana Pandey, Balgovind Tiwari, Amber Kumar, Keerti Swarnkar, Danish Javed, Prateek Behera

**Affiliations:** 1 Department of AYUSH, All India Institute of Medical Sciences (AIIMS), Bhopal, India; 2 Government Ayurvedic College, Atarra, Banda, Uttar Pradesh, India; 3 Department of Paediatrics, All India Institute of Medical Sciences (AIIMS), Bhopal, India; 4 Department of Paediatrics, All India Institute of Medical Sciences (AIIMS), Bhopal, India; 5 Department of AYUSH, All India Institute of Medical Sciences (AIIMS), Bhopal, India; 6 Department of Paediatric orthopaedics, All India Institute of Medical Sciences (AIIMS), Bhopal, India

**Keywords:** Spastic diplegic cerebral palsy (CP), *Swarna Prashan*, panchakarma

## Abstract

Spastic cerebral palsy is a leading cause of childhood disability, with spastic diplegia being one of the most common subtypes and multidisciplinary approach in integrated form will be useful for reducing spasticity. Integrative intervention may provide less harmful and easy mode of treatment for CP children. Therefore, it is of interest to observe the effect of Ayurvedic treatment with *Swarna Prashan* on reducing spasticity in children with spastic diplegic cerebral palsy. An observational pre-post study was conducted at AIIMS Bhopal over 12 weeks and the total duration of study was 12 months. Twenty children aged 6 months to 10 years with pre-diagnosed spastic diplegic cerebral palsy (Modified Ashworth Scale ≤ 3) were included. Interventions comprised of oral *Swarna Prashan* and Panchakarma therapy (*Mahanarayan* and *Mahamaash oil* massage) every 2 weeks for 12 weeks. Spasticity (Modified Ashworth Scale), gross motor function (GMFCS-E & R) and anthropometric parameters were recorded pre- and post-intervention.

## Background:

Cerebral Palsy is the most common cause of neurodevelopmental abnormalities in children. Recent research studies report that the prevalence of cerebral palsy ranges from 1 to nearly 4 per 1,000 live births of children. Research study Patel *et al.* evaluate that cerebral palsy is a neuro-motor disorder characterized by abnormal movement, posture and tone. Research studies shows that cerebral palsy has multiple associated conditions and complexities that need continuous support by a multidisciplinary management team [1, 2]. Ayurvedic medicine describes various modalities for improving neuromuscular function and reducing spasticity. *Swarna Prashan* (a traditional formulation containing purified gold) has been used to enhance immunity, neurological development and cognitive function [3]. Various studies show effect of Panchakarma therapies such as medicated oil massage (*Abhyanga*) and medicated rice bolus fomentation (*Shashtika shali pinda sweda*) in spastic cerebral palsy and these have been reported to improve muscle tone and functional performance [4, 5]. Various paediatric diseases are described in classical Ayurvedic literature books under the title of Bal Roga [6, 7]. The safety and efficacy of suvarna prashan in pediatric practice are well described by acharya kashyap in the kashyap samhita, a classical text of ayurveda. Research study shows that the Ayurvedic treatment modality is effective in reducing the disability in children with spastic cerebral palsy [8, 9]. Panchakarma therapy is effective in improving growth and development by improving head holding and sitting. It reduces the spasticity of the lower and upper limbs in patients. Cerebral Palsy has multiple associated symptoms other than movement that need to be managed with adulthood [10]. Ayurvedic medicine, as an Integrative approach, has the potential to improve the quality of life and disease symptoms in children with cerebral palsy [11, 12, 13]. Therefore, it is of interest to evaluate the effect of Ayurveda treatment modalities on spasticity and Gross motor function outcomes in children with spastic diplegic cerebral palsy.

## Materials and Methods:

## Patient involvement:

Pre-diagnosed cases of spastic Cerebral Palsy who fulfilled the inclusion criteria attending the OPD of the department of PMR, Orthopedic, Pediatrics and the department of AYUSH, AIIMS Bhopal were registered, after taking informed consent from parents/guardians, for the present study. The study was approved by the Institutional Ethics Committee (IHEC-LOP/2022/IL078).

## Trial design:

The study was prospective open level interventional single group observational study with pre- and post-test design.

## Trial setting and sample selection:

Children who fulfilled the inclusion criteria will be included in this study from the OPD of department of PMR, Orthopaedic, Paediatrics and the department of AYUSH, AIIMS Bhopal. Ayurvedic treatment will be given in Ayurveda unit, Department of AYUSH, AIIMS Bhopal.

## Eligibility criteria:

Pre-diagnosed cases of spastic CP who fulfilled the inclusion criteria from the age of 6 months to 10 years with irrespective of sex are eligible for this study.

## Inclusion criteria:

[1] Pre-diagnosed cases of Spastic Diplegia CP

[2] Age >6 months to 10 years both male and female children

[3] Modified Ashworth Scale value is <3 or =3

## Exclusion criteria:

[1] Children with other types of CP like Spastic hemiplegia or quadriplegia, hypotonic, dyskinetic and athetoid.

[2] Children with major congenital disorders.

[3] Children with any systemic diseases that may turn out to be hindrance during the course of the study.

[4] Children with other diseases such as diabetes, acute infection, etc.

[5] Modified Ashworth Scale value is 4.

## Intervention:

## Oral medication:

*Swarna Prashan* was administered for 2 weeks at the interval of 2 weeks.

Total 3 times provide the oral medication for 12 weeks at the interval of 2-2 weeks.

Swarna bhasm dose was as per AFI/ as calculated with young's formula based on adult dose with honey and ghee ratio will be 2:1 ratio.

## Form of medicine:

Medicine will be administered in paste form.

## Time of administration:

30 minutes before or after feed, Daily single dose ready to use for 2 weeks.

## Panchakarma therapy:

Sarvanga *Abhyanga* and *Shastika Shali Pinda Sveda* apply for the next 2 week before the *Swarna Prashan*. Total 3 times apply externally for 12 weeks at the interval of 2-2 weeks before taking *Swarna Prashan*. Sarvanga *Abhyanga* with *MahanarayanTaila* (oil) and *Mahamaash tail (oil)* followed by Svedana (fomentation) with *Shastika Shali Pinda Sveda* (for about 45 min) as per classical method.

## Srah Pichu:

Srah Pichu application with *Bala tail* (oil) will be applicable continue for 12 weeks. Total treatment duration was for 12 weeks with two follow up on 8 weeks and 12 weeks during treatment.

## Study duration:

The study was completed in 12 months.

## Outcome measures:

[1] Anthropometrical measurement

[2] Developmental milestone

[3] Modified Ashworth Scale (MAS)- to assess muscle spasticity (Table 1 - see PDF).

[4] Gross Motor Function classification system -Expanded and Revised (GMFCS-E & R) - to assess gross motor functions

## Gross Motor Function Classification System Expanded and Revised (GMFCS-E & R):

GMFCS-E & R is used to assess gross motor function. It is especially used to check the ability to walk, for children from 2 to 18 years of age of CP. It is used both for self-initiated movements or movements by any devices such as walkers, crutches, canes or wheelchairs etc. It emphasizes especially on abilities of children not only their limitations (Table 2 - see PDF).

It shows the significant result (P <0.05) for weight and highly significant for height (P=0.001). It shows significant results (P < 0.05) in limb spasticity through spasticity grading (MAS) whereas GMFCS-E & R shows insignificant results (P>0.05) (Table 3 - see PDF).

## Assessment of the total effect of therapy:

[1] Maximum Improvement: >75% improvement.

[2] Moderate Improvement: >50-75% improvement.

[3] Mild Improvement: >25-50% improvement.

[4] No Improvement: ≤25% improvement.

## Sample size:

This is a pilot study so minimum 20 children were registered as convenient sampling method for this study.

## Statistical analysis plan:

Statistical analysis was done by using paired t test for pre and post treatment comparisons. Descriptive statistics were used to summarize result of this pilot study.

## Observations:

[1] A Total of 20 patients were registered, of whom 16 were male children and 4 were female children.

[2] 18 children belonged to the 0-5 year's age group and 2 children belonged to the 6-10 years age group.

[3] 6 children had Microcephaly, but 14 had normal head circumference.

[4] All 20 children had gross motor delay.

## Primary outcome:

As shown in Table 3 (see PDF), Significant reverse reduction in MAS scores post-treatment (p < 0.05)

## Secondary outcomes:

GMFCS scores non-significant in the majority of children. Anthropometric measures showed minor improvements in weight and height (Table 3 - see PDF).

## Functional Improvement:

Most participants achieved >25% functional improvement.

## Results and Discussion:

In majority of patients, total effect was found approx. 25% which shows significant result (P<0.05). As this disorder is incurable, this percentage of improvement also helps the patient to improve QOL. Treatment of this kind of condition is just like a pyramid, if we are able to make small improvement in an earlier age than it will reflect its major benefit in later age in the form of developing skills. This study will be helpful to evaluate efficacy of ayurvedic treatment in spastic cerebral palsy. *Swarna Prashan* may be helpful to improve cognitive and intellectual activity of children of cerebral palsy. Intervention of panchakarma and *Swarna Prashan* in early age may be useful to provide proper growth at proper time for physical, mental, intellectual and neurodevelopmental growth in cerebral palsy patients. The protocol of the proposed pilot research study conducted at department of AYUSH, AIIMS Bhopal as tertiary care centre. This pilot study is designed to investigate whether it is feasible, effective and safe to use ayurvedic panchakarma therapy with *Swarna Prashan* for pediatric cerebral palsy patients with standard conventional rehabilitation treatment. The study provides useful information for integrating ayurvedic treatment into rehabilitation care of spastic diplegic cerebral palsy as part of the effort of integrating ayurveda traditional medicine in hospitals. Research study shows the use and effectiveness of ayurveda treatment should be considered as a part of intervention to reduce spasticity outcomes in children with spastic cerebral palsy [14]. Various study focused on the beneficial use of integrative practices especially in pediatric health issues that may be effective from their application [15]. These are focused on Effectiveness of various Ayurveda treatment modalities in the management of spasticity in children with cerebral palsy. Various oral Ayurvedic medicines and different panchakarma therapy like *basti*, *abhayang*, *swedan etc.*, are used to reduce spasticity in cerebral palsy patients [16, 17- 18]. Various research studies show the effect of different mode of complementary treatment to evaluate effect of traditional massage and *basti* *etc.*, studies tried to describe pharmacodynamic understanding of alternative treatments as contemporary approach for including these as a part of integrative medicine. These studies evaluate the effect of various type of massage on spastic cerebral palsy and determine the prevalence of massage use among children with cerebral palsy [19, 20-21]. Cerebral palsy is considered as *Vata Vyadhi* in ayurveda. It is characterized by *Dhatu Kshaya Lakshana* with the manifestation of vitiated *Vata*. *Snehan* and Swedan are effective for vitiated *Vata*. In which *MahanarayanTaila* (oil) is *Vatat shamak* and *Mahamaash tail* (oil) followed by Svedana (fomentation) with *Shastika Shali* Pinda Sveda are worked as *Brinhan* and *Balya* property. The safety and efficacy of Suvarna Prashan in pediatrics practice, are well described in the classical texts of Ayurveda. It is the ancient method that is used to provide a good life to the children with physical, mental, intellectual and social health. So, Ayurvedic treatment helps the children to grow up with better physical and mental strength as well as intellectual performance. It also helps to improve Immunity, which may be helpful to reduce co-morbidities in the children of CP and also helpful to improve concentration, memory and cognitive and intellectual skills in children. Intervention of Sarvanga *Abhyanga* with *MahanarayanTaila* (oil) and *Mahamaash tail* (oil) followed by Svedana (fomentation) with *Shastika Shali Pinda Sved* with Srah Pichu application by *Bala tail* (oil) and *Swarna Prashan* in early age may be useful to provide proper growth at proper time for physical, mental, intellectual and neurodevelopmental growth in cerebral palsy patients. *Pravartaka Cheshtanam* (normal movement of joints) is difficult by increased resistance with passive stretch and velocity dependent due to Spasticity and asymmetric joints in children with spastic cerebral palsy. It is caused by vitiated *Vata dosha* and *Vata* cannot perform its normal function. Research study shows relevance use of *Abhyanga* (massage) specially in CP patients. Panchakarma helped in reduction of vitiated *Vata dosha* by the process of *Srotoshodhana*. Sarvang *Abhyanga* by *MahanarayanTaila* (oil) and *Mahamaash tail* (oil) followed by *Svedana* (hot fomentation) with *Shastika Shali* Pinda Sveda and Sirah Pichu application with *Bala tail* (oil) works on *Vata* resides in Sparshnendriya which is placed in *Tvacha* and these therapies might work directly on vitiated *Vata dosha* to bring it back to prakrit state with proper functioning. Panchakarma therapy with *Swarna Prashan* acts on central nervous system by stimulating enteric nervous system. Spasm was reduced significantly by this integrative intervention in this study. Regular monitoring of cerebral palsy patients is very necessary to examine mobility and ability for performing daily activities. Due to elderly caregivers and failing social services, children of spastic cerebral palsy face challenge and they have decrease ability to access adaptive devices [22, 23]. The first and foremost aim of the management of cerebral palsy is not cure but to improve their capabilities with cognitive and intellectual skills so that they can survive with good quality of life in their life span. It may reduce the psychological and economic burden of caregivers too. This study shows efficacy of ayurvedic treatment with *Swarna Prashan* in spastic cerebral palsy. Ayurvedic treatment with *Swarna Prashan* may be helpful to reduce spasticity and improve cognitive and intellectual activity of Children of cerebral palsy. This is the time to develop new integrative approaches in health sector especially for our country India which has large demographic diversity with big population. For standardization of integrated treatment, it is necessary to do further research with large population in future research.

## Conclusion:

Ayurvedic treatment modalities show potential as supportive interventions with standard conventional treatment in the management of spastic diplegic cerebral palsy. Integrating these traditional therapies into multidisciplinary rehabilitation programs may enhance patient outcomes. It may be helpful to develop new integrative approaches in the health sector for developing a multi-disciplinary approach to improve the quality of life as well as rehabilitation programs in CP patients.

## Funding:

This research study was conducted under Intra Mural research Project Scheme of AIIMS, Bhopal.

## Institutional human ethics committee:

Ethical approval was obtained from Institutional Human Ethics Committee of institute AIIMS Bhopal Ref.no. IHEC-LOP/2022/IL078.

## Informed consent statement: 

Informed consent was obtained from parent of all subjects involved in the study for participating in this study as well as to publish this paper.
